# Visual and equipment-free reverse transcription recombinase polymerase amplification method for rapid detection of foot-and-mouth disease virus

**DOI:** 10.1186/s12917-018-1594-x

**Published:** 2018-08-31

**Authors:** Libing Liu, Jinfeng Wang, Ruoxi Zhang, Mi Lin, Ruihan Shi, Qingan Han, Jianchang Wang, Wanzhe Yuan

**Affiliations:** 1 Center of Inspection and Quarantine, Hebei Entry-Exit Inspection and Quarantine Bureau, Shijiazhuang, 050051 People’s Republic of China; 2 College of Veterinary Medicine, Agricultural University of Hebei, No.38 Lingyusi Street, Baoding, Hebei 071001 People’s Republic of China; 3 Hebei Animal Disease Control Center, Shijiazhuang, 050050 People’s Republic of China; 4 Hebei Academy of Science and Technology for Inspection and Quarantine, Shijiazhuang, 050051 People’s Republic of China; 5 State Key Laboratory of Veterinary Etiological Biology, Lanzhou Veterinary Research Institute, Chinese Academy of Agricultural Sciences, Lanzhou, 730046 People’s Republic of China

**Keywords:** FMDV, 3D gene, RPA, LF probe, Lateral flow strip

## Abstract

**Background:**

Foot-and-mouth disease (FMD), which is caused by foot-and-mouth disease virus (FMDV), is a highly contagious tansboundary disease of cloven-hoofed animals and causes devastating economic damages. Accurate, rapid and simple detection of FMDV is critical to containing an FMD outbreak. Recombinase polymerase amplification (RPA) has been explored for detection of diverse pathogens because of its accuracy, rapidness and simplicity. A visible and equipment-free reverse-transcription recombinase polymerase amplification assay combined with lateral flow strip (LFS RT-RPA) was developed to detect the FMDV using primers and LF probe specific for the 3D gene.

**Results:**

The FMDV LFS RT-RPA assay was performed successfully in a closed fist using body heat for 15 min, and the products were visible on the LFS inspected by the naked eyes within 2 min. The assay could detect FMDV serotypes O, A and Asia1, and there were no cross-reactions with vesicular stomatitis virus (VSV), encephalomyocarditis virus (EMCV), classical swine fever virus (CSFV), porcine reproductive and respiratory syndrome virus (PRRSV), porcine circovirus 2 (PCV2) and pseudorabies virus (PRV). The analytical sensitivity was 1.0 × 10^2^ copies in vitro transcribed FMDV RNA per reaction, which was the same as a real-time RT-PCR. For the 55 samples, FMDV RNA positive rate was 45.5% (25/55) by LFS RT-RPA and 52.7% (29/55) by real-time RT-PCR. For the LFS RT-RPA assay, the positive and negative predicative values were 100% and 80%, respectively.

**Conclusions:**

The performance of the LFS RT-RPA assay was comparable to real-time RT-PCR, while the LFS RT-RPA assay was much faster and easier to be performed. The developed FMDV LFS RT-RPA assay provides an attractive and promising tool for rapid and reliable detection of FMDV in under-equipped laboratory and at point-of-need facility, which is of great significance in FMD control in low resource settings.

## Background

Foot-and-mouth disease (FMD) is a highly contagious viral disease of wild and domesticated cloven-hoofed animals. The causative agent, foot-and-mouth disease virus (FMDV), a non-enveloped, single-stranded positive-sense RNA virus, belongs to the genus *Aphthovirus* within the family *Picornaviridae* [1]. FMDV exists in seven distinct serotypes comprising O, A, C, Asia1 and South African Territories (SAT) serotypes SAT1, SAT2 and SAT3 and multiple subtypes due to the high mutational rate of the virus [1]. Although the mortality rate of FMD is generally low, the disease can be economically devastating due to production losses in endemic countries and trade restrictions in FMD-free countries. It is estimated that annual global impact of FMD in endemic regions alone is between US$ 6.5 and 21 billion [2].

The above facts clearly indicate that the early, rapid and robust diagnosis of FMD is imperative in the prevention and control of the disease. FMD is characterized by vesicular lesions and ulcerations on the tongue, mouth, nasal region and coronary bands of infected animals [3]. Nevertheless, reliable diagnosis based on clinical signs alone can sometimes be difficult because the clinical signs are often mild in adult sheep and goats [4] and a number of viral diseases clinically mimic FMD, including vesicular stomatitis (VS), swine vesicular disease (SVD), vesicular exanthema of swine (VES), and Senecavirus A (SVA) infection. Therefore, laboratory diagnostic tools for detection of FMDV are imperative for the effective control and elimination of the disease. Currently, several conventional methods are available for the detection of FMDV, including virus isolation (VI), antigen-capture ELISA (Ag-ELISA), and immunochromatographic lateral flow device (Ag-LFD) [5, 6]. VI is a relatively laborious and time-consuming method that must be performed in a high-containment biosafety laboratory. Ag-ELISA has a limited sensitivity and also requires skilled technicians to perform and interpret the assays. Ag-LFD has only been validated for use with epithelial samples [5]. Molecular diagnostic assays are now recognized as reliable detection methods for FMDV. A number of reverse transcription polymerase chain reaction (RT-PCR) assays have been reported and accepted widely for the detection of FMDV RNA, such as RT-PCR and real-time RT-PCR [7–9]. The RT-PCR assays are designed for use in well-equipped laboratories with reliable electrical supply and highly trained technicians, and unsuitable for being used in under-equipped laboratories and in field. Although several real-time RT-PCR assays have been transferred onto a portable platform and trialled successfully in field settings [7, 10, 11], expensive high precision instrumentation and consistent electrical power are still needed. When compared to current RT-PCR assays, the use of isothermal technologies reduces the need for high precision instrumentation, consistent electrical power and complex sample preparation [12]. Recently, several field-deployable isothermal DNA amplification assays including the reverse transcription insulated isothermal PCR (RT-iiPCR), reverse transcription loop-mediated isothermal amplification (RT-LAMP), nucleic acid sequence based amplification (NASBA) and reverse transcription helicase dependent amplification (RT-HDA) have been developed for FMDV detection [13–16]. However, RT-LAMP assay requires six primers and has unsatisfactory reliability in detection of highly variable viruses [15, 17], which makes the assay difficult to design for FMDV. Furthermore, the results are usually produced within 60 min–120 min for the above methods, and depend on water baths and specialized instruments [13–16]. Therefore, a simple, rapid, and sensitive method is still needed for the point-of-need (PON) detection of FMDV.

**Fig. 1:**
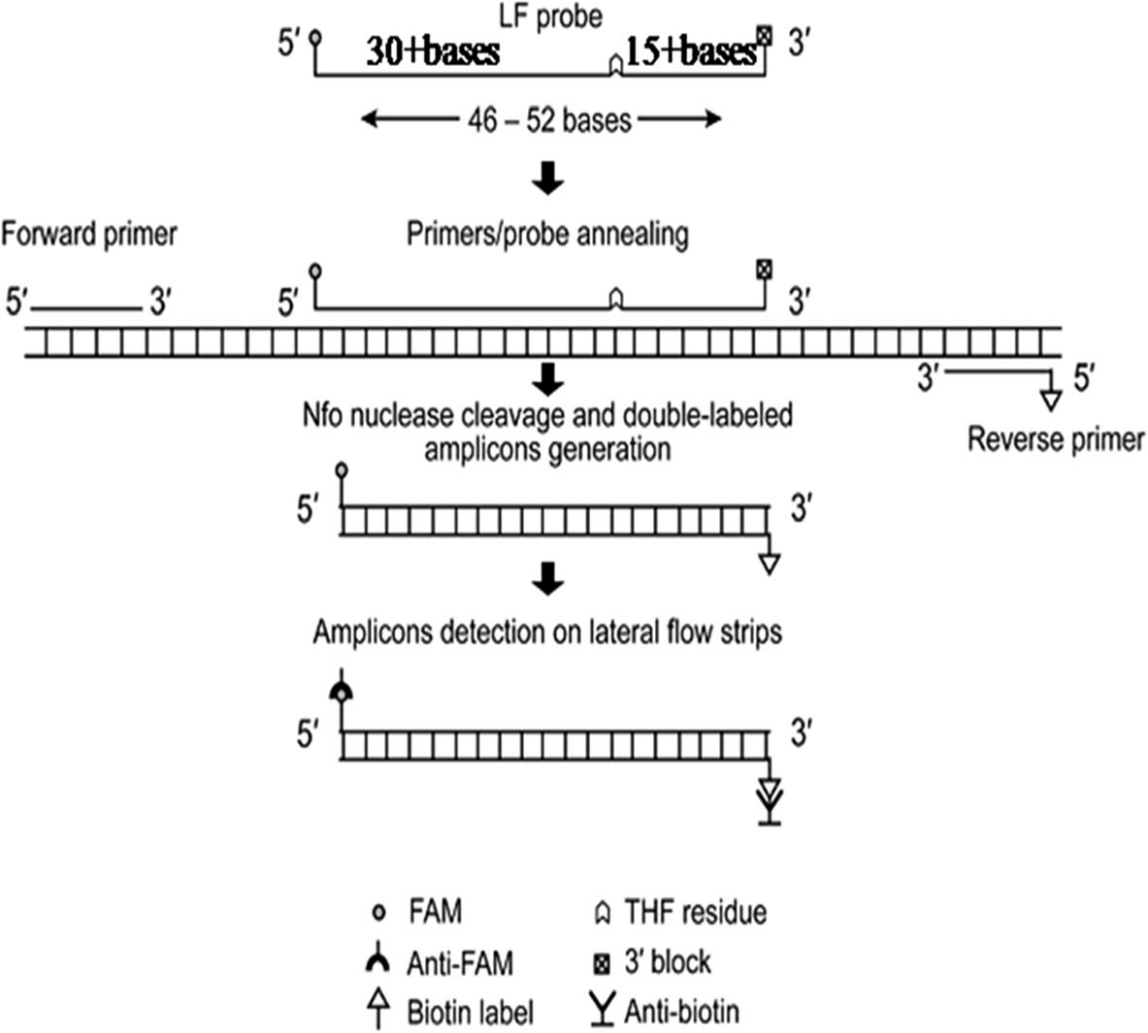
Diagram of LF probe and post-RPA detection with lateral flow strip. LF probe is typically 46–52 nucleotides long, at least 30 of which are placed 5′ to the THF site, and at least a further 15 nucleotides are located 3′ to the site. Detection of amplicons is accomplished by capture of tags with anti-FAM antibodies and biotin-ligand molecules generating a visual colored line on LFS

As an isothermal DNA amplification technique, recombinase polymerase amplification (RPA) has been demonstrated to be rapid, specific, sensitive, and cost-effective, and has been applied widely in the detection of different pathogens [18, 19]. Recombinase, single-stranded DNA-binding protein (SSB) and strand-displacing polymerase are three core enzymes employed in RPA. Recombinases form complexes with primers and pair the primers with homologous sequences in the template DNA. SSB binds to the displaced strand and stabilizes the resulting loop, then DNA amplification is initiated by DNA polymerase [18, 19]. Abd El Wahed et al. had developed a real-time RT-RPA assay based on exo probe for rapid detection of FMDV, while the assay still depended on the specialized instrument, ESEQuant tubescanner [20]. With the Endonuclease IV, LF probe and the reverse primer labelled at the 5′ end with a biotin in the RPA reaction system, the products could be detected by the naked eye. The LF probe oligonucleotide backbone includes a 5′- FAM group, an internal tetrahydrofuran residue (THF) and a 3′- C3-spacer (Fig. 1) [18, 19]. The generated amplicons dual labelled with FAM and Biotin are then detected by the naked eye in ‘sandwich’ assay formats, such as the lateral flow strip (LFS) that contains anti-FAM gold conjugates and biotin-ligand molecules. A series of LFS RPA assays have been developed for the detection of porcine parvovirus (PPV), peste des petits ruminants virus (PPRV), infectious bovine rhinotracheitis virus (IBRV) and bovine ephemeral fever virus (BEFV) [21–24].

In this study, we developed an equipment-free RPA assay for rapid, specific and sensitive detection of FMDV, which was combined with a LFS (USTAR, Hangzhou, China) and performed by incubating the reactions tubes in a closed fist using body heat.

## Methods

### Virus strains

Different serotypes of foot-and-mouth disease virus (FMDV) and a panel of other pathogens considered dangerous to pigs were used in the study. Denatured cell-free extracts of FMDV (serotype O, A and Asia1) were obtained from the commercial Liquid-phase Blocking ELISA Kit (Lanzhou Veterinary Research Institute, Lanzhou, China). Encephalomyocarditis virus (EMCV, strain BD2), porcine circovirus 2 (PCV2, strain HB-MC1), and the viral RNA of vesicular stomatitis virus (VSV) were maintained in our laboratory. Porcine reproductive and respiratory syndrome virus (PRRSV, strain JXA1-R, Pulike Biological Engineering), classical swine fever virus (CSFV, strain AV1412, Ringpu) and pseudorabies virus (PRV, strain Barth-K61, Ringpu) were from commercial attenuated live vaccines.

### Clinical and spiked samples

Twelve RNA extracted from the vesicular fluid and epithelium tissue collected from pigs experimentally infected with FMDV serotype O were provided by the State Key Laboratory of Veterinary Etiological Biology (Animal Ethics Committee of the Lanzhou Veterinary Research Institute, approval number: LVRIAEC 2012–018) and used for the clinical validation of the FMDV LFS RT-RPA assay. Twenty serum samples were collected from clinically healthy pigs and twenty serum samples were collected from clinically healthy cattle, which were tested to be FMDV RNA negative by a real-time RT-PCR [7]. Eight swine and eight bovine sera were spiked with the denatured cell-free extracts of FMDV serotype O at the ratio of 1:1, 1:10, 1:20, 1:40, 1:80, 1:100, 1:200 and 1:400, respectively, and the other sera were used as control samples. The above samples and denatured cell-free extracts of FMDV serotype O, A and Asia1 were used as the clinical, spiked and control samples in this study.

### DNA/RNA extraction

FMDV, EMCV, PRRSV and CSFV viral RNA was extracted using Trizol Reagent (Invitrogen, Waltham, USA), PRV and PCV2 viral DNA was extracted using the TIANamp Virus DNA kit (Tiangen, Beijing, China), which were performed according to manufacturer’s instructions, respectively. Two hundred μL of the sera and FMDV type O were used for viral RNA extraction using the Trizol Reagent, and viral RNA was finally eluted in 20 μL of nuclease-free water. Viral RNA and DNA were quantified using a ND-2000c spectrophotometer (NanoDrop, Wilmington, USA). All RNA and DNA templates were stored at − 80 °C until use.

### Generation of FMDV standard RNA

The 1104 bp RT-PCR product covering the 3D gene of FMDV was generated from viral genomic RNA of FMDV serotype O using 3D-F and 3D-R primers (Table 1). In vitro transcribed FMDV standard RNA was generated using the 3D gene RT-PCR products as described previously [25], and diluted in ten-fold series to achieve RNA concentrations ranging from 1.0 × 10^7^ to 1.0 × 10^0^ copies/μL.

**Table 1:** Sequences of primers and probes for FMDV RT-PCR, real-time RT-PCR and LFS RT-RPA assays

Assay	Primers and probes	Sequence 5´-3´	Amplicon size (bp)	References
RT-PCR	3D-F	CCCATTGAGTATCTACGAGG	1104	This study
	3D-R	CAACGCAGGTAAAGTGATC		
real-time	FMDV-F	ACTGGGTTTTACAAACCTGTGA	86	[7]
RT-PCR	FMDV-R	GCGAGTCCTGCCACGGA		
	FMDV-P	FAM-TCCTTTGCACGCCGTGGGAC-BHQ1		
LFS	FMDV-LFS-F	TTGGTCACTCCATTACCGATGTCACTTTCCTC	258	This study
RT-RPA	FMDV-LFS-R	5’-Biotin-AACGCAGGTAAAGTGATCTGTAGCTTGGAAT		
	FMDV-LFS-P	5’-FAM-GCACGCCGTGGGACCATACAGGAGAAGTT		
		GAT(THF)TCCGTGGCAGGACTCG-C3-spacer-3’		

### RPA primers and LF probe

The nucleotide sequences of 3D gene are highly conserved among the different serotypes of FMDV, and the 3D gene was chosen as the target of the RPA. According to the reference sequences of different FMDV strains (accession numbers: serotype O: KF985189; KX712091; NC_004004; HQ412603; JX947859; serotype A: HQ832592; KJ968663; KU127247; serotype Asia I: KC412634; DQ533483; HQ8322592; serotype C:FJ824812;DQ409191; serotype SAT1: KU821590; JF749860; serotype SAT2: JF749862; JX014256; KU821592; serotype SAT3: KM268901; KJ820999), the primers and LF probe were designed based on 3D gene. Primers and LF probe’s specificity was also tested in silico with the nucleotide sequence of other picornaviruses, such as VSV (accession numbers: NC_001560, MF196237), SVDV (accession numbers: AF268065, EU151461), VESV (accession numbers: NC_002551, KM26948, U76874), and SVA (accession numbers: NC_011349, DQ641257, KC667560, KR063107). The primers and LF probe were listed in Table 1 and synthesized by a commercial company (Sangon Biotech Co., Shanghai, China).

### LFS RT-RPA

LFS RT-RPA reactions were performed in a 50 μL volume containing 29.5 μL rehydration buffer and 2.5 μL magnesium acetate (280 mM) from the TwistAmp™ nfo kit (TwistDX, Cambridge, UK). Other components included 420 nM each RPA primer (FMDV-LFS-F and FMDV-LFS-R), 120 nM LF probe (FMDV-LFS-P), 200 U MMLV reverse transcriptase (Takara, Dalian, China), 40 U Recombinant RNase Inhibitor (Takara, Dalian, China) and 1 μL of viral RNA or 5 μL of sample RNA. Except for the viral template and magnesium acetate, the other reagents were prepared in a master mix and distributed into a 0.2 mL freeze-dried reaction tube containing a dried enzyme pellet. One μL of viral RNA and 2.5 μL of magnesium acetate were pipetted into the tubes. The RPA was performed in the technician’s closed fist at room temperature for 5, 10, 15 and 20 min as described previously [26, 27]. The RPA products, which were dual labelled with FAM and Biotin, were detected using LFS as described previously [26, 27]. A testing sample was considered positive when both the test line and the control line were visible, negative when only the control line was visible, and invalid when the control line was invisible.

### Analytical specificity and sensitivity analysis

Ten ng of RNA or DNA was used as template for the analytical specificity analysis of the LFS RT-RPA assay. The assay was evaluated against a panel of pathogens considered dangerous to pigs, FMDV serotype O, A, Asia1, VSV, PRRSV, CSFV, EMCV, PRV and PCV2. Three independent reactions were performed by three different technicians in the laboratory, office or in the field with an ambient temperature of 23.8 °C, 23.0 °C and 19.3 °C, respectively.

The ten-fold serial diluted in vitro transcribed RNA with concentrations ranging from 1.0 × 10^7^ to 1.0 × 10^0^ copies/μL were used as the standard RNA for FMDV LFS RT-RPA assay. One μL of each dilution was then amplified by the LFS RT-RPA to determine the limit of detection (LOD) of the assay. Three independent reactions were performed by three different technicians.

### Validation with the clinical, spiked and control samples

The LFS-RPA method was assessed on clinical samples from experimentally infected pigs, bovine and porcine serum samples spiked with FMDV serotype O, and control samples from clinically healthy cattle and pigs. All samples tested by LFS RT-RPA were also tested by a real-time RT-PCR [7]. Positive predictive (the probability that the disease is present when the test is positive) and negative predictive (the probability that the disease is absent when the test is negative) values were calculated for the LFS RT-RPA and real-time RT-PCR. Since the status of the clinical, spiked and control samples were known, FMDV LFS RT-RPA and real-time RT-PCR results were classified as true positive (TP) or true negative (TN) if in agreement with the known status of tested samples. If results differed from the known status of tested samples, they were classified as false positive (FP) or false negative (FN). Positive predictive value was calculated as TP/(TP + FP) and negative predictive value as TN/(TN + FN) and expressed as a percentage.

## Results

### Optimization of the reaction time

**Fig. 2:**
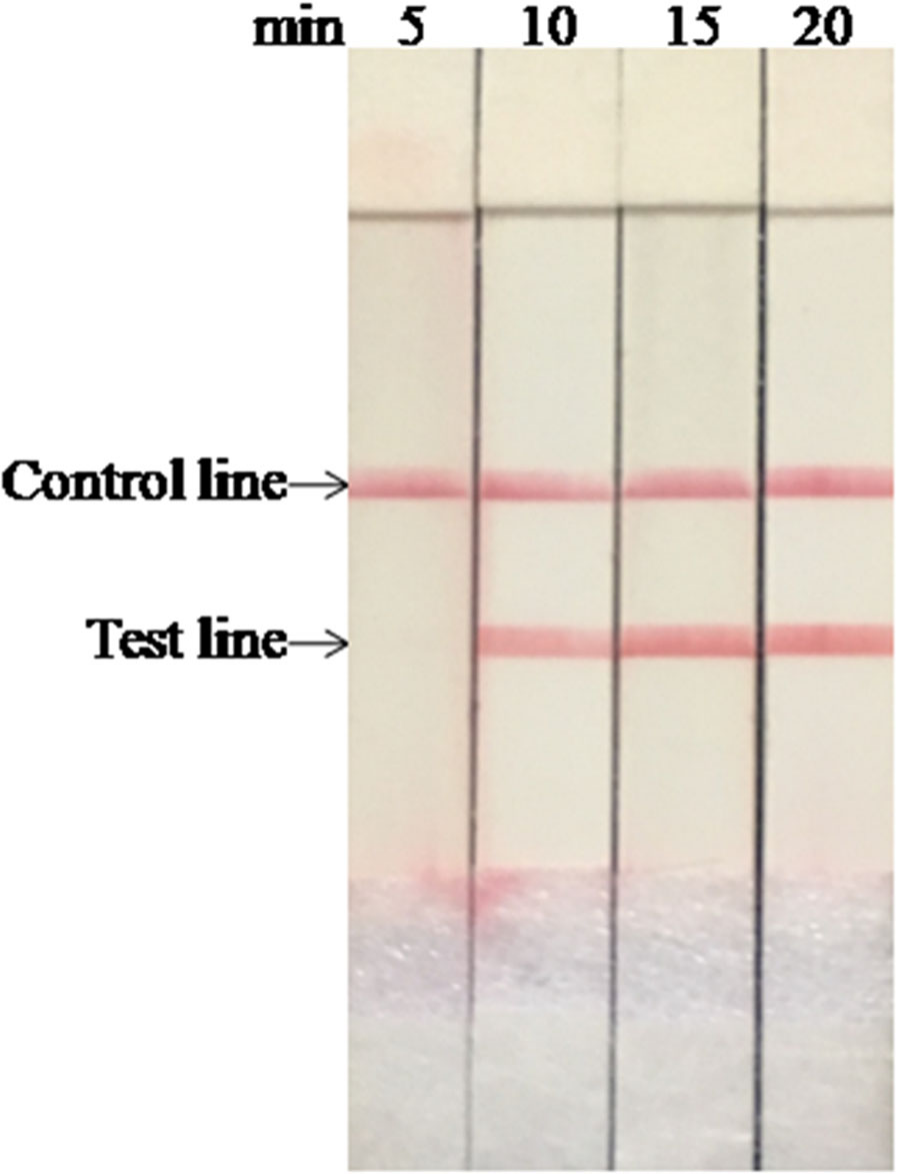
Determination of FMDV LFS RT-RPA reaction time. The test line was visible when the amplification time was longer than 10 min

The results from performing the LFS RT-RPA test with different reaction times are shown in Fig. 2. No amplified products were observed in reactions incubated for 5 min and slightly weak amplified product observed at 10 min. When the incubation time increased over 15 min, the assay performance was improved, and there were no clear differences between 15 and 20 min. Similar results were observed in three independent reactions, and the temperature in the closed fists was 35.8 °C, 36.7 °C and 35.7 °C, respectively. Therefore, 15 min was set as the optimal incubation time for FMDV LFS RT-RPA assay.

### Analytical specificity and sensitivity

**Fig. 3:**
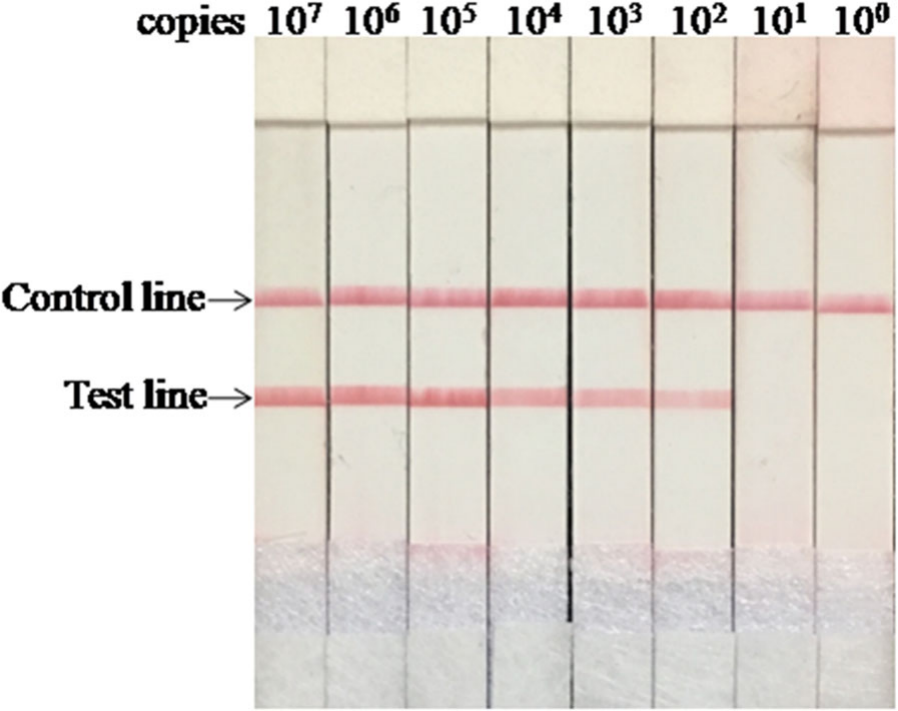
Analytical sensitivity of FMDV LFS RT-RPA assay. The LOD of the assay was 1.0 × 10^2^ copies per reaction

Using 10 ng of viral RNA and DNA as template, the results showed only the FMDV serotype O, A and Asia1 were detected by LFS RT-RPA while the other viruses were not detected (Fig. 3). No cross-detections were observed, which showed the high analytical specificity of the assay. Three independent reactions were performed with similar results, demonstrating the good repeatability of the assay.

The level of detection was 1.0 × 10^2^ copies as shown in Fig. 3. The results were similar from all three technicians. The LOD was the same as that of the real-time RT-PCR.

### Evaluation of LFS RT-RPA with the clinical and spiked samples

Ten out of 12 clinical samples were FMDV RNA positive in the LFS RT-RPA (Table 2). For the 16 spiked samples, 12 samples (the spiked swine and bovine sera from 1:1 to 1:100) were FMDV RNA positive in LFS RT-RPA, while 14 samples (the spiked swine and bovine sera from 1:1 to 1:200) were FMDV RNA positive in real-time RT-PCR (Table 2). At the dilution of 1:200, all spiked samples were negative in LFS RT-RPA, while they were positive in real-time RT-PCR with the Ct values of 37.15 and 37.64, respectively (Table 2). The spiked sera at the dilution 1:400, and the 24 negative control sera were all negative in both assays (Table 2). The denatured cell-free extracts of FMDV serotype O, A and Asia1 were positive in both LFS RT-RPA and real-time RT-PCR, with the Ct values of 18.95, 20.63 and 20.37, respectively (Table 2). The positive predicative values for the LFS RT-RPA and real-time RT-PCR were 100%, and the negative predicative values for the LFS RT-RPA and real-time RT-PCR were 80% and 92.3%, respectively. It took less than 20 min in the LFS RT-RPA assay to obtain the positive results, while it took 30–51 min in the real-time RT-PCR with the Ct values ranging from 18.95 to 37.64. These results indicated that the performance of the LFS RT-RPA assay was comparable to real-time RT-PCR, while the LFS RT-RPA assay was faster.

**Table 2:** Comparison of FMDV LFS RT-RPA with real-time RT-PCR assays performed on RNA extracts from the virus strains, clinical samples, spiked serum samples and samples from healthy controls

Sample type	Sample name	LFS RT-RPA	real-time RT-PCR(Ct)
Virus strains	FMDV type O	+	18.95
	FMDV type A	+	20.63
	FMDV type Asia1	+	20.37
Clinical	4	+	23.45
samples	28	+	32.58
	124	+	26.78
	125	+	30.42
	126	+	30.24
	131	+	24.26
	133	+	30.76
	140	+	33.75
	208	+	25.11
	209	-	35.85
	213	-	36.96
	217	+	32.10
Spiked	Swine serum 1	+	23.47
serum samples	(1:1)		
	Swine serum 2	+	25.75
	(1:10)		
	Swine serum 3	+	26.41
	(1:20)		
	Swine serum 4	+	28.45
	(1:40)		
	Swine serum 5	+	33.24
	(1:80)		
	Swine serum 6	+	34.45
	(1:100)		
	Swine serum 7	-	37.15
	(1:200)		
	Swine serum 8	-	>40.00
	(1:400)		
	Bovine serum 1	+	23.08
	(1:1)		
	Bovine serum 2	+	25.51
	(1:10)		
	Bovine serum 3	+	26.64
	(1:20)		
	Bovine serum 4	+	29.56
	(1:40)		
	Bovine serum 5	+	34.40
	(1:80)		
	Bovine serum 6	+	35.14
	(1:100)		
	Bovine serum 7	-	37.64
	(1:200)		
	Bovine serum 8	-	>40.00
	(1:400)		
Control samples	Swine serum	-	>40.00
	(9-20)		
	Bovine serum	-	>40.00
	(9-20)		

+ : positive; - : negative

## Discussion

Outbreaks of FMD have caused great economic losses to the livestock farming worldwide, therefore, accurate and rapid diagnosis is imperative for the prevention and control of the disease. Although RT-PCR assays have played an important role in the control of FMD and have been accepted widely for the detection of FMDV in the laboratories, it still needs a lengthy process for the clinical samples being transported to laboratories in suitable cold-chain conditions, which could impose delays on diagnosis and consequently on critical decision making. The PON molecular diagnostic assays for FMDV would be of significant importance for the disease control.

This study describes a visible, equipment-free LFS RT-RPA assay with high sensitivity and specificity for rapid detection of FMDV. The FMDV LFS RT-RPA reaction tubes were held in a closed fist for 15 min, and the results were inspected directly by the naked eyes within 2 min. FMDV serotype C, SAT1–3, SVDV, VESV and SVA were not included in the analytical specificity analysis, which is a shortcoming of this study. RPA is tolerant to 5–9 mismatches in primer and probe showing no influence on the performance of the assay [20, 28], and the maximum number of mismatches found within one sequence was four in some FMDV serotypes available in GenBank (e.g. accession numbers DQ533483, HQ412603, KU821590, JF749862, KJ820999, and KT968663). It is assumed the assay would detect all the seven serotypes of FMDV, based on the facts that the LFS RT-RPA assay targets the conserved 3D gene of FMDV and that the in silico analysis of the primers and probe shows their high specificity for FMDV.

RPA operates at a wide range of temperatures, and does not require the reaction temperature to be precisely controlled [19]. TwistDx recommends an incubation temperature of 37 °C (the temperature of the human body), others studies have shown that RPA retains reliable functionality between 31 °C and 43 °C [22, 29, 30], even between 30 °C and 45 °C [22, 30]. Our previous study also showed that RPA could work well for detection of PCV2 between 34 °C and 42 °C [31]. Normal human body temperature (36.1–37 °C) is within the above temperature range, and several RPA assays had been developed to perform the reaction using body heat either holding in the axilla or in closed fists [32, 33]. In this study, FMDV LFS RT-RPA assay was performed by holding the reaction tubes in the closed fists, which is one feature of the assay.

Most of the published LFS RT-RPA assays are either developed for DNA or performed using water baths [21–23, 29, 30, 33]. In the PPRV and BEFVLFS RT-RPA assays, the viral RNA was reverse transcribed to cDNA firstly, then the viral cDNA but not viral RNA was used as the template [21, 24]. In this assay, the MMLV (4 U/μL) and RNase inhibitor (0.8 U/μL) were added into the RPA reaction system and the LFS RT-RPA worked well with FMDV RNA as the template directly, which is the other feature of the assay.

The LFS RT-RPA assay demonstrated the same positive predicative value as the real-time RT-PCR, while the negative predicative of the LFS RT-RPA (80%) was lower than real-time RT-PCR (92.3%). For two clinical samples and two spiked samples, the testing results were FMDV RNA positive in the real-time RT-PCR, while negative in the LFS RT-RPA. The sensitivity of the LFS RT-RPA was lower than the real-time PCR, nevertheless, the assay showed distinct advantages in other respects, especially the detection time and equipment requirement. Although the above results are inspiring, the assay should still be validated by analysis of more FMDV RNA positive clinical samples.

As in the real-time RT-PCR, RNA extraction is necessary in the LFS RT-RPA in this study. One of the main reasons for developing such assay is its potential use in the field or, at least, in the absence of a reliable power supply. Presently, the cost per reaction performed in FMDV LFS RT-RPA and real-time RT-PCR are approximately $9.8 and $4.8, respectively. While considering no requirement of any incubation instruments, the rapidness of the reaction, the LFS RT-RPA is still a very promising tool in the FMD control. With the offering of TwistAmp® Liquid RPA kits and the wide application of RPA technology, the cost would be further reduced and the RPA would be closer to become a true PON isothermal molecular assay.

## Conclusions

A rapid, visible and equipment-free method using body heat is developed successfully for PON diagnosis of FMD. The good specificity, sensitivity, and easy sample-to-answer protocol make the developed LFS RT-RPA assay ideal for the accurate and rapid detection of FMDV RNA in under-equipped laboratory and at PON facility, especially in low resource settings.

## Abbreviations

CSFV: Classical swine fever virus
CT: Cycle threshold
EMCV: Encephalomyocarditis virus
FMDV: Foot-and-mouth disease virus
LFS: Lateral flow strip
MMLV: Moloney Murine Leukemia virus
PCV2: Porcine circovirus 2
PON: Point-of-need
PRRSV: Porcine reproductive and respiratory syndrome virus
PRV: Pseudorabies virus
RPA: Recombinase polymerase amplification
SVA: Senecavirus A
TT: Threshold time
VSV: Vesicular stomatitis virus
